# Preclinical evaluation of dasatinib, a potent Src kinase inhibitor, in melanoma cell lines

**DOI:** 10.1186/1479-5876-6-53

**Published:** 2008-09-29

**Authors:** Alex J Eustace, John Crown, Martin Clynes, Norma O'Donovan

**Affiliations:** 1 National Institute for Cellular Biotechnology, Dublin City University, Dublin 9, Ireland; 2 Dept of Medical Oncology, St Vincent's University Hospital, Dublin 4, Ireland

## Abstract

**Background:**

Metastatic melanoma is a highly chemotherapy resistant tumour. The use of newer targeted therapies alone and in combination with chemotherapy may offer new hope of improving response to treatment. Dasatinib, a multi-target kinase inhibitor, is currently approved for the treatment of chronic myeloid leukaemia and has shown promising results in preclinical studies in a number of solid tumours.

**Methods:**

We examined the effects of dasatinib on proliferation, chemo-sensitivity, cell cycle arrest, apoptosis, migration and invasion in human melanoma cell lines. Expression and activation of Src kinase, FAK and EphA2 were also examined in the melanoma cells.

**Results:**

Dasatinib inhibited growth of three of the five melanoma cell lines. Comparison with sorafenib showed that in these three cell lines dasatinib inhibited growth at lower concentrations than sorafenib. Dasatinib in combination with the chemotherapy drug temozolomide showed greater efficacy than either drug alone. Dasatinib induced cell cycle arrest and apoptosis and significantly inhibited cell migration and invasion of melanoma cells. Dasatinib inhibition of proliferation was associated with reduced phosphorylation of Src kinase, while decreased phosphorylation of FAK was implicated in dasatinib-mediated inhibition of migration and invasion in melanoma cells.

**Conclusion:**

Dasatinib has both anti-proliferative and anti-invasive effects in melanoma cells and combined with chemotherapy may have clinical benefit in the treatment of malignant melanoma.

## Background

Metastatic melanoma is notoriously resistant to cytotoxic chemotherapy. Commonly used agents such as dacarbazine and temozolomide yield poor response rates of less than 20% [1] and combination regimes have not been proven superior over single agents [2]. Therefore novel, more efficacious treatment strategies are urgently needed for melanoma.

Sorafenib (BAY43-9006) inhibits vascular endothelial growth factor receptor (VEGFR) and Raf kinase, but also has activity against c-kit and platelet derived growth factor receptor beta (PDGFR-β). Activating B-Raf mutations are detected in greater than 60% of malignant melanomas [3] and sorafenib inhibits the growth of melanoma cells carrying B-Raf mutations. Sorafenib has shown little activity as a single agent in the treatment of malignant melanoma, irrespective of B-Raf status [4], however in combination with carboplatin it has shown promising clinical activity [5] and is presently being tested in several clinical trials in melanoma either alone or in combination with other agents .

Src kinase regulates key pathways in metastasis including cell adhesion, invasion and motility [6] and members of the Src family have been implicated in melanoma progression [7-11]. Both Src and Yes are reported to be elevated in melanoma cells compared to normal melanocytes [7,12]. Dasatinib, a multi-target tyrosine kinase inhibitor, targets Src kinase, in addition to BCR-Abl, c-KIT, PDGFR and ephrin-A receptor kinases. It is the most potent Src kinase inhibitor currently in clinical development with an IC_50 _of 0.5 nM for Src kinase (IC_50 _of < 30 nM for the other targets) [13]. Dasatinib has shown preclinical activity in prostate cancer [14], triple negative breast cancer [15] and colon cancer cells.

Due to the deficiency of effective treatment options for advanced melanoma and the reported relationship between Src kinase and melanoma progression, we examined the preclinical activity of Src inhibition, using dasatinib, alone and in combination with temozolomide in metastatic melanoma cell lines.

## Methods

### Cells and reagents

Lox-IMVI, Malme-3M, Sk-Mel-5, and Sk-Mel-28 were obtained from the Department of Developmental Therapeutics, National Cancer Institute (NCI) and HT144 from the American Tissue Culture Centre (ATCC). Cell lines were grown at 37°C with 5% CO_2 _in RPMI medium with 10% FCS (Gibco) except HT144 which was grown in McCoys 5A (Sigma-Aldrich) with 10% FCS. Stock solutions of temozolomide (9.7 mM), (Department of Developmental Therapeutics, National Cancer Institute), epirubicin (3.45 mM), taxotere (11.6 μM) (Dept of Pharmacy, St. Vincent's University Hospital), dasatinib (10 mM), sorafenib (10 mM) (Sequoia Research Products) and imatinib (16.9 mM) (Novartis) were prepared in dimethyl sulfoxide (Sigma-Aldrich).

### Preparation of cell extracts for Western blotting

500 μL RIPA buffer with 1 × protease inhibitors, 2 mM PMSF and 1 mM sodium orthovanadate (Sigma-Aldrich) was added to cells and incubated on ice for 20 minutes. Following centrifugation at 10,000 rpm for 5 minutes at 4°C the resulting lysate was stored at -80°C. Protein quantification was performed using the Bicinchoninic acid (BCA) assay (Pierce). 40 μg of protein in sample buffer was heated to 95°C for 5 minutes and proteins were separated on 7.5 or 10% gels (Cambrex). The protein was transferred to Hybond-ECL nitrocellulose membrane (Amersham Biosciences). The membrane was blocked with blocking solution (PBS + 0.1% Tween + 5% skimmed milk powder (BioRad)) at room temperature for 1 hour, then incubated overnight at 4°C with 1 μg/ml primary antibody (mouse anti-Epha2, Millipore; mouse anti-Src kinase, Upstate Cell Signalling Solutions; rabbit anti-phospho-Src py 418, Biosource Europe; mouse anti-FAK kinase BD Biosciences; rabbit anti-FAK py 861 and py 397, Invitrogen; mouse anti-tubulin, Sigma-Aldrich) in blocking solution. The membrane was washed three times with PBS-Tween, then incubated at room temperature with anti-mouse secondary antibody (Sigma-Aldrich) at 1:1000 dilution or anti-rabbit secondary antibody (Pierce) at 1:3000 dilution) in blocking solution for 1 hour. The membrane was washed three times with PBS-Tween followed by one PBS wash. Detection was performed using Luminol (Santa Cruz Biotechnology). For detection of phosphorylated EphA2, EphA2 was immunoprecipitated from 500 μg of protein using EphA2 antibody (Millipore) and immunoblotted with a mouse anti-phosphotyrosine antibody (Upstate Cell Signalling Solutions).

### Proliferation assay

Proliferation was measured using an acid phosphatase assay. 1 × 10^3 ^cells/well were seeded in 96-well plates, apart from HT144 and Malme-3M which were seeded at 2 × 10^3 ^cells/well. Plates were incubated overnight at 37°C followed by addition of drug at the appropriate concentrations and incubated for a further 5 days until wells were 80% to 90% confluent. All media was removed and the wells were washed once with PBS. Paranitrophenol phosphate substrate (0.263 g of PNP in 100 ml sodium acetate buffer) was added to each well and incubated at 37°C for 2 hours. 50 μl of 1 M NaOH was added and the absorbance was read at 405 nM (reference – 620 nM), as previously described [16].

### Invasion assays

Invasion and migration assays were performed as previously described [17], using 1 × 10^5 ^cells in matrigel-coated 24-well invasion inserts for invasion assays and uncoated inserts for migration assays. Cells were incubated for 6 hours before dasatinib treatment to allow cells to attach and then incubated at 37°C with dasatinib at varying concentrations for 24 hours. Cells were stained with crystal violet and the number of invading/migrating cells was estimated by counting 10 fields of view at 200 × magnification. The average count was multiplied by the conversion factor 140 (growth area of membrane divided by field of view area, viewed at 200 × magnification) to determine the total number of invading/migrating cells. All assays were performed in triplicate.

### Terminal DNA transferase-mediated dUTP nick end labelling (TUNEL) assay

2.5 × 10^4 ^cells were seeded per well in 24-well plates and incubated overnight at 37°C, followed by addition of drug at the appropriate concentrations. After 72 hours, media was collected and the wells washed once with PBS. Cells were trypsinised and added to the media collected for each sample. Cells were centrifuged at 300 × g for 5 minutes and the media was aspirated. 150 μl of PBS was added, the pellet re-suspended and the total volume transferred to a round bottomed 96 well plate. 50 μL of 4% para-formaldehyde was added to the wells and mixed. Cells were incubated at 4°C for 60 minutes. The plate was centrifuged at 300 × g for 5 minutes and the supernatant aspirated leaving approximately 15 μL in each well. The remaining volume was used to resuspend the cells and 200 μL of ice cold 70% ethanol was added to the cells. The plates were then stored at -20°C for 2 hours. After fixing the cells were stained according to the protocol for the TUNEL assay (Guava Technologies). Cells were analysed on the Guava EasyCyte (Guava Technologies). Positive and negative controls were performed with each assay.

### Cell cycle assays

2.5 × 10^4 ^cells were seeded per well in 24-well plates and incubated overnight at 37°C. After 24 hours cells were synchronised by removing the media and replacing it with serum free medium (SFM) for a further 24 hours. SFM was removed and the cells incubated for a further 6 hours in media containing serum before the drug was added at the appropriate concentrations. Plates were then incubated at 37°C for a further 24 hours. Media was collected and the wells washed once with PBS. Cells were trypsinised and added to the media collected for each sample. Cells were centrifuged at 300 × g for 5 minutes and the media was aspirated. 150 μl of PBS was added, the pellet re-suspended and the total volume transferred to a round bottomed 96 well plate. The plate was centrifuged at 300 × g for 5 minutes and the supernatant aspirated leaving approximately 15 μL in each well. The remaining volume was used to resuspend the cells and 200 μL of ice cold 70% ethanol was added. The plates were then stored at -20°C for 2 hours. After fixing the cells were stained according to the protocol for the Guava Cell Cycle assay (Guava Technologies). Cells were analysed on the Guava EasyCyte and the data was analysed using Modfit LT software (Verity).

### Statistical analysis

IC_50 _values were calculated using CalcuSyn software (BioSoft). For Lox-IMVI, combination index (CI) values were calculated using CalcuSyn software. A CI value of < 1 is considered synergistic, 1 is considered additive and > 1 is considered antagonistic. CI values were not calculated for the other cell lines, as dasatinib did not achieve 50% inhibition of growth at concentrations up to 1 μM. The Student's *t *test was used to compare temozolomide IC_50_s alone and in combination with dasatinib, migration/invasion assays and cell cycle assays *P *< 0.05 was considered statistically significant. ANOVA one way analysis was performed to compare dasatinib alone, taxotere/epirubicin alone and the combination. *P *< 0.05 was considered statistically significant.

## Results

### Sensitivity to dasatinib

**Figure 1:**
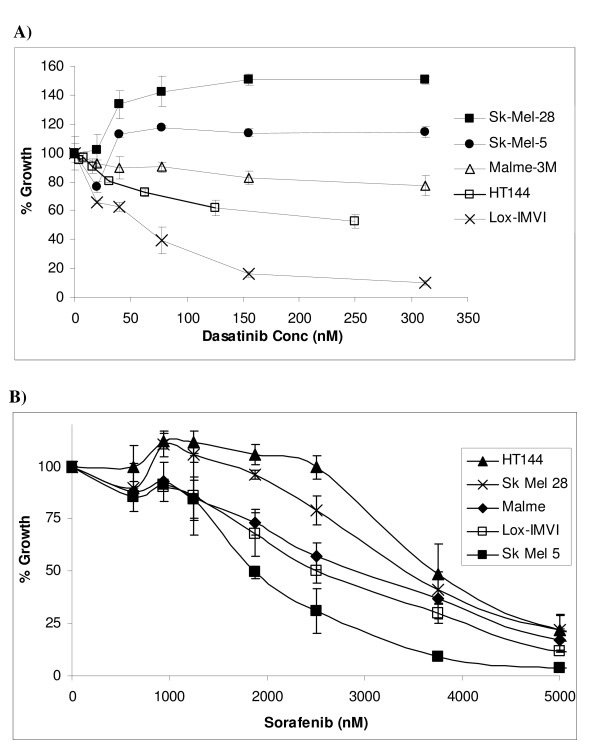
**Percentage growth inhibition by A) dasatinib and B) sorafenib in a panel of melanoma cell lines.** Error bars represent the standard deviation of triplicate experiments.

The effect of dasatinib on proliferation was tested in a panel of five melanoma cell lines (Figure 1). Lox-IMVI displays the greatest sensitivity to dasatinib with an IC_50 _of 35.4 nM (± 8.8 nM). HT144 and Malme-3M also display some sensitivity to dasatinib with a maximum growth inhibition of 40% and 30%, respectively, achieved in these cell lines at 1 μM dasatinib. Growth of Sk-Mel-28 and Sk-Mel-5 appear to be slightly increased in response to dasatinib treatment. IC_50 _values for sorafenib ranged from the most sensitive cell line Sk-Mel-5 (IC_50 _= 1.4 ± 0.4 μM) to the most resistant HT144 (IC_50 _= 4.1 ± 0.4 μM). Sensitivity to the multi-target kinase inhibitor, imatinib, was also examined in HT144 and Lox-IMVI cells. Imatinib did not inhibit the growth of either cell line at concentrations up to 5 μM (See additional file 1: Effect of imatinib on proliferation).

### Dasatinib in combination with chemotherapy

**Figure 2:**
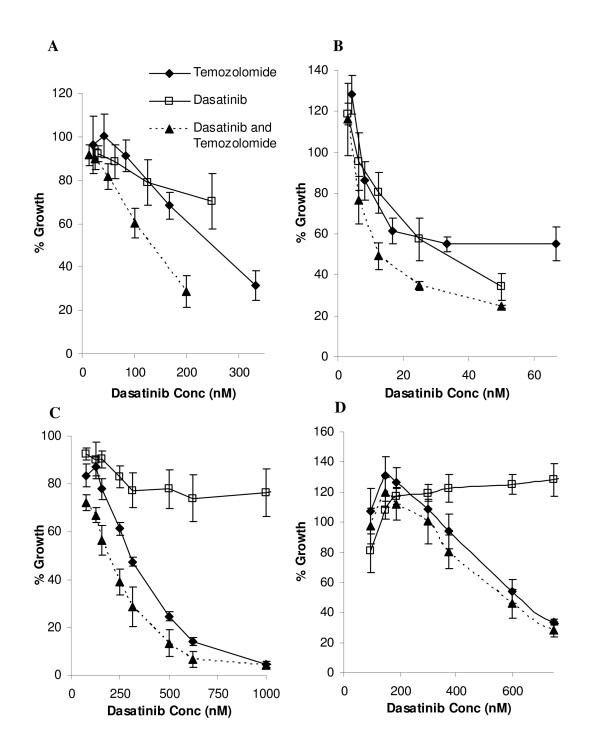
**Combination assays testing dasatinib with temozolomide at the specified ratios in (A) HT144 (ratio 1:1500), (B) Lox-IMVI (ratio 1:3000), (C) Malme-3M (ratio 1:800) and (D) Sk-Mel-28 (ratio 1:800) cells. **Concentrations of temozolomide are represented as a ratio of the dasatinib concentration. Error bars represent the standard deviation of triplicate experiments.

The effect of dasatinib in combination with chemotherapy was examined in the three dasatinib responsive cell lines, Lox-IMVI, HT144 and Malme-3M and in one of the dasatinib-resistant cell lines, Sk-Mel-28. In both HT144 and Malme-3M, dasatinib enhanced response to temozolomide (Figure 2). In Lox-IMVI, CI values (CI value at ED_50 _= 0.88 ± 0.03) revealed the combination of dasatinib and temozolomide was slightly synergistic. The IC_50 _for temozolomide when administered in combination with dasatinib, was significantly reduced compared to temozolomide alone in HT144 (227 μM versus 359 μM, p = 0.038) and in Malme-3M (212 μM versus 343 μM, p = 0.024). In Sk-Mel-28, which is resistant to dasatinib, temozolomide combined with dasatinib produces a similar response to temozolomide alone (See Additional file 2: Temozolomide IC_50_s).

The effects of dasatinib in combination with epirubicin and taxotere were also examined in HT144 and Lox-IMVI (See additional file 3: Combination assays of dasatinib with epirubicin or taxotere). In both HT144 and Lox-IMVI, dasatinib combined with epirubicin increased inhibition of proliferation compared to either drug alone. The combination of taxotere and dasatinib also significantly increased inhibition of proliferation compared to either drug alone.

### Effect of dasatinib on apoptosis and cell cycle arrest

**Figure 3:**
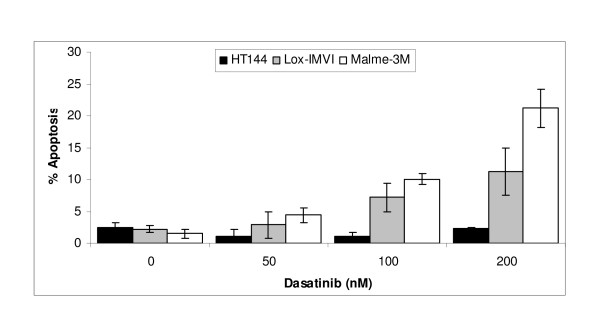
Measurement of dasatinib induced apoptosis in HT144, Lox-IMVI and Malme-3M using the TUNEL assay.

In Lox-IMVI and Malme-3M cells, increasing concentrations of dasatinib induced apoptosis (Figure 3). However, in HT144 cells dasatinib does not appear to induce apoptosis with concentrations up to 200 nM. Dasatinib treatment resulted in a slight increase in G1 arrest in HT144 (p = 0.07) and a significant increase in Lox-IMVI (p = 0.0045), compared to control untreated cells (Table 1). Dasatinib did not induce cell cycle arrest in Sk-Mel-28 or Malme-3M cells (See additional file 4: Effect of dasatinib on cell cycle arrest).

**Table 1:** Percentage of cells in the G1 phase of the cell cycle, in control and dasatinib treated samples.

**Cell Lines**	**Control**	**50 nM Dasatinib**	**100 nM Dasatinib**	**200 nM Dasatinib**
**HT144**	**51.6** ± *4.5*	**58.3** ± *1.2*	**59.6** ± *2.1*	**59.4** ± *6.1*
**Lox-IMVI**	**35.4** ± *3.0*	51.0 * ± *4.6*	56.6 * ± *4.6*	53.0 * ± *6.6*
**Malme-3M**	**71.7** ± *1.5*	**70.2** ± *4.0*	**70.9** ± *2.0*	**69.5** ± *2.4*
**Sk-Mel-5**	**57.6** ± 1.6	**55.3** ± 2.2	**57.6** ± 3.3	**58.9** ± 4.3

Note: '*' indicates p < 0.05.

### Effect of dasatinib on invasion and migration

**Figure 4:**
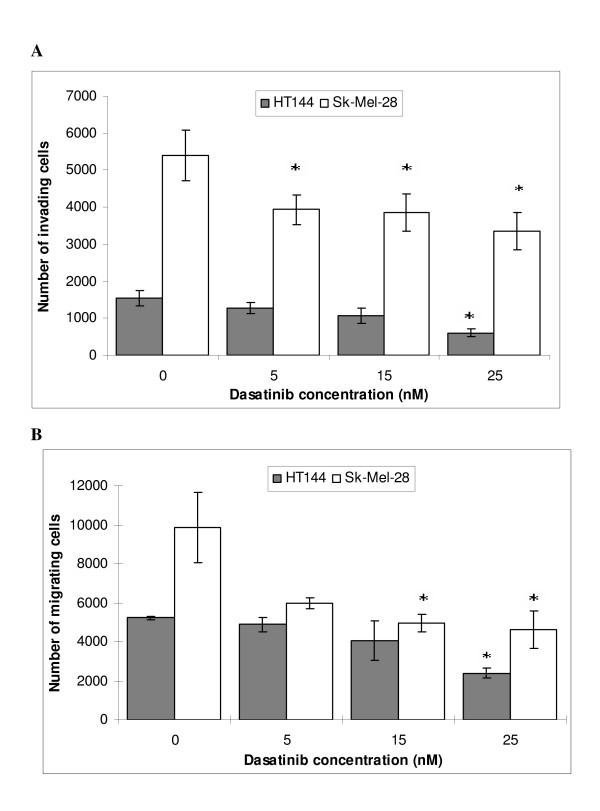
**Effect of dasatinib on (A) invasion and (B) migration in HT144 and Sk-Mel-28 melanoma cell lines. **Error bars represent the standard deviation of triplicate assays. '*' indicates p < 0.05.

The effects of dasatinib on invasion and migration were examined in two invasive cell lines, one dasatinib sensitive (HT144) and one resistant cell line (Sk-Mel-28). Dasatinib significantly decreased invasion of HT144 and Sk-Mel-28 cells (25 nM dasatinib: HT144 p = 0.05; Sk-Mel-28 p = 0.016) (Figure 4A) and migration of both cell lines (25 nM dasatinib: HT144 p = 0.001; Sk-Mel-28 p = 0.019) (Figure 4B). The concentrations of dasatinib used in the invasion/migration assays were non-toxic to the cells (data not shown).

### Effect of dasatinib on Src kinase, EphA2 and FAK

**Figure 5:**
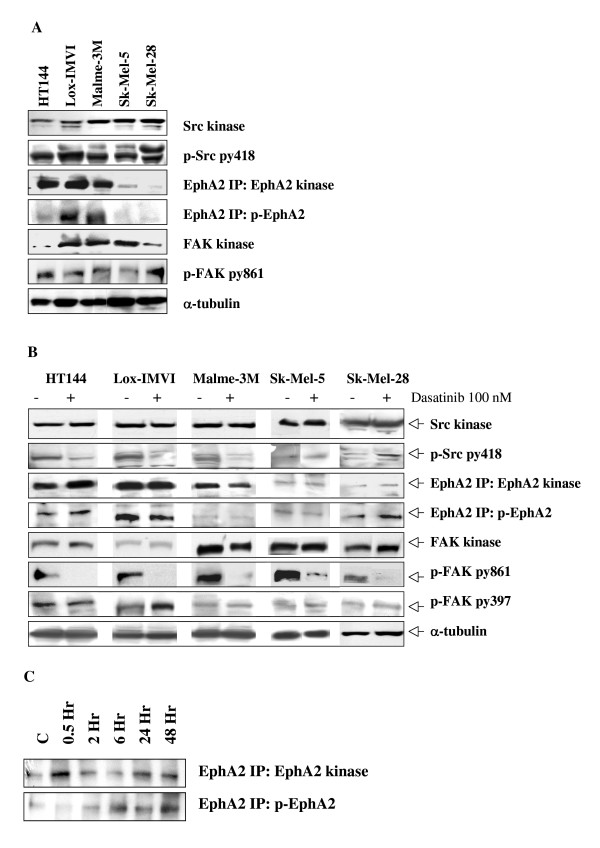
**Western blotting for Src kinase, phospho-Src kinase py 418, FAK, phospho-FAK py 397 and py 861, immunoprecipitated (IP) EphA2, IP phospho-EphA2 and α-tubulin in (A) the panel of melanoma cell lines; and (B) HT144, Lox-IMVI, Malme-3M, Sk-Mel-5 and Sk-Mel-28 untreated (control) or treated with 100 nM dasatinib for 6 hours.** (C) Western blotting for IP EphA2, IP phospho-EphA2 in Lox-IMVI untreated (control) and treated with 100 nM dasatinib for up to 48 hours.

Src, EphA2, FAK and phosphorylated Src, EphA2 and FAK were detected in all cell lines tested, although the levels of phosphorylated Src kinase detected were low (Figure 5A). Phosphorylation of Src was decreased in HT144, Lox-IMVI and Malme-3M in response to dasatinib treatment (Figure 5B), but the level of Src phosphorylation appeared to be slightly increased in Sk-Mel-28 cells treated with dasatinib (Figure 5B). EphA2 phosphorylation was unchanged in all cell lines tested, after 6 hours of treatment with 100 nM dasatinib. In Lox-IMVI cells treated with 100 nM dasatinib for up to 48 hours, EphA2 phosphorylation was transiently reduced after 30 minutes but activation was restored by 2 hours. Phospho-FAK py861 was reduced in all cell lines tested after treatment with dasatinib whereas phospho-FAK py397 was unaffected by treatment with dasatinib.

## Discussion

We have evaluated the effects of dasatinib, a multi-targeted tyrosine kinase inhibitor, in human melanoma cell lines [6]. In a previous study in breast cancer cell lines, sensitivity to dasatinib was characterised as greater than 60% inhibition, moderate sensitivity as 40–59% inhibition and resistance as less than 40% inhibition in response to 1 μM dasatinib [15] (assuming higher concentrations would not be achievable *in vivo*) [15]. Therefore, Lox-IMVI can be classified as being highly sensitive to dasatinib, HT144 moderately sensitive and the remaining three cell lines are resistant, although Malme-3M shows some sensitivity.

Sorafenib which is currently in clinical trials for advanced melanoma, has shown little activity when tested alone but shows promising results when tested in combination with chemotherapy [5]. In the five cell lines tested in this study, which are B-Raf mutated , the IC_50 _for sorafenib was above 1 μM in each case. These results suggest that dasatinib-sensitive melanoma cells are more sensitive to dasatinib than to sorafenib *in vitro*.

Furthermore, dasatinib in combination with temozolomide significantly improved response in HT144 and Lox-IMVI compared to either drug alone. In Malme-3M cells, there was a small but significant improvement in response compared to temozolomide alone. In the dasatinib-resistant cell line Sk-Mel-28, the combination was slightly better than temozolomide alone although the difference was not significant. Therefore the combination of dasatinib with temozolomide may improve response in some melanoma patients. In dasatinib resistant tumours, the addition of dasatinib would not impact on sensitivity to temozolomide but may help to prevent further tumour spread by inhibiting melanoma cell migration and invasion, as we observed in dasatinib-resistant Sk-Mel-28 cells.

Studies in lung cancer [18], head and neck squamous cell carcinoma [19] and malignant pleural mesothelioma [20] showed that dasatinib induces both cell cycle arrest and apoptosis. In Lox-IMVI, the most sensitive cell line, treatment with dasatinib induced both apoptosis and cell cycle arrest. In the other dasatinib responsive cell lines, HT144 and Malme-3M, dasatinib induced either cell cycle arrest or apoptosis respectively. Therefore, optimal response to dasatinib in melanoma cells may require efficient induction of both cell cycle arrest and apoptosis.

Imatinib targets Bcr-Abl, c-Kit and PDGFR. Previous studies identified that c-kit expression was reduced with melanoma progression and trials testing imatinib as a single agent showed no benefit in the clinical setting [21,22]. However recent studies have identified a group of chronic sun damaged patients who maintain c-kit expression despite melanoma progression [23] and as a result clinical trials have been undertaken to target c-kit with imatinib in this population [21].

Imatinib however does not inhibit the growth of either HT144 or Lox-IMVI cells. Thus sensitivity of melanoma cell lines to dasatinib may be due to targeting Src kinase or EphA receptors, which are not targeted by imatinib. Differences in the level or phosphorylation of Src kinase do not appear to predict sensitivity to dasatinib in the melanoma panel. Similar to preclinical studies in other solid tumour types [20], phosphorylation of Src was reduced in dasatinib sensitive cell lines, whereas in the dasatinib resistant cell lines Sk-Mel-28 and Sk-Mel-5, phospho-Src was either unchanged or slightly increased, in response to dasatinib treatment. Thus inhibition of Src phosphorylation may be an appropriate marker of response to dasatinib. Serrels *et al *[24] showed that inhibition of phospho-Src in peripheral blood mononuclear cells correlated with inhibition of phospho-Src in colon tumours. Measuring changes in phospho-Src in peripheral blood mononuclear cells may therefore serve as a surrogate marker for response to dasatinib in the clinic [25].

Previous studies have shown that dasatinib treatment did not reduce phosphorylation of FAK at Tyr397, an autophosphorylation site required for recruitment of Src kinase which in turn phosphorylates FAK at Tyr576, Tyr577, and Tyr861 [24]. Phosphorylation at these sites is important for FAK downstream signalling [26]. Dasatinib reduced the level of FAK phosphorylation at Tyr861 in all of the melanoma cell lines and therefore does not appear to be associated with inhibition of proliferation but may play a role in inhibition of migration and invasion in melanoma cells. In colon cancer cells, reduced phosphorylation of FAK at tyrosine 861 was implicated in dasatinib-mediated inhibition of migration and invasion [24]. Recently enzyme assays have shown that dasatinib is a potent inhibitor of several additional kinases, including FAK (IC_50 _= 0.2 nM) [27]. Therefore, dasatinib may directly target FAK, independently of Src, resulting in inhibition of migration/invasion without inhibition of proliferation, as was observed in Sk-Mel-28 cells.

Other dasatinib preclinical studies did not examine the role of EphA receptors in response to dasatinib. EphA2 has been identified as a potential dasatinib sensitivity biomarker [28]. Interestingly EphA2 levels were significantly higher in the three dasatinib sensitive cell lines than in the two resistant cell lines. Although the number of cell lines is small, this suggests that EphA2 expression may predict response to dasatinib treatment and warrants further investigation in a larger panel of cell lines. Dasatinib treatment for 6 hours had no effect on phosphorylation of EphA2. However, in Lox-IMVI, phosphorylation of EphA2 was transiently decreased at 30 minutes, but was restored by 2 hours. EphA2 activity may also be altered by decreased phosphorylation of Src and FAK, which form a complex with EphA2 [29]. Dasatinib may also target other members of the Ephrin receptor family such as EphB4 [27]. Further research is required to elucidate the role of Ephrin receptors in response to dasatinib treatment in melanoma and other solid tumours.

The *in vitro *effects of dasatinib in melanoma cell lines observed in this study provide strong evidence for evaluation of dasatinib in clinical trials in melanoma patients. Two clinical trials of dasatinib in melanoma are currently underway, including a phase I/II study of dasatinib in combination with dacarbazine .

## Conclusion

Our preclinical evaluation of dasatinib, shows that it has anti-proliferative, pro-apoptotic and anti-invasive effects in some melanoma cells *in vitro*. Furthermore, combining dasatinib with temozolomide improved response in melanoma cell lines. Thus, dasatinib is an exciting new therapeutic option for malignant melanoma. Phospho-Src represents a promising pharmacodynamic marker for response to dasatinib and high levels of EphA2 may be a predictive marker for dasatinib. Identification and validation of appropriate biomarkers will be crucial to maximise the potential clinical benefits of dasatinib treatment for melanoma.

## Competing interests

The authors declare that they have no competing interests.

## Authors' contributions

AJE contributed to the design of the study and carried out the proliferation assays, TUNEL assays, cell cycle assays, Western blotting and statistical analysis. JC and MC contributed to the interpretation of the data. NOD conceived the study, supervised the research, and participated in interpretation of the data and drafting the manuscript. All authors read and approved the final manuscript.

## Supplementary Material

Additional file 1 Effect of imatinib on proliferation. The data compares the effect of imatinib on the proliferation of HT144 and Lox-IMVI. Click here for file

Additional file 2 Comparison of IC50 concentrations of temozolomide when tested alone and in combination with dasatinib in HT144, Lox-IMVI, Malme-3M and Sk-Mel-28 cells. Standard deviations represent average results of triplicate experiments. IC50 values were compared using the Student's T-test. Click here for file

Additional file 3 Combination assays of dasatinib with epirubicin or taxotere in HT144 and Lox-IMVI. Click here for file

Additional file 4 Effect of dasatinib on cell cycle arrest. Comparing the effect of dasatinib versus untreated cells on the percentage of cells tested in the G1, S and G2/M phases of cell cycle. Click here for file
