# Supplementary material for: Preclinical evaluation of dasatinib, a potent Src kinase inhibitor, in melanoma cell lines
Source: J Transl Med. 2008 Sep 29;6:53. doi: 10.1186/1479-5876-6-53 (PMC2569026; doi:10.1186/1479-5876-6-53)
Supplement: Additional file 1 — Effect of imatinib on proliferation. The data compares the effect of imatinib on the proliferation of HT144 and Lox-IMVI. [file 1479-5876-6-53-S1.doc]

Additional file 1: Proliferation assay showing percentage growth of HT144 and Lox-IMVI cells treated with 1, 2 and 5 µM imatinib, relative to the control.
