# Supplementary material for: Preclinical evaluation of dasatinib, a potent Src kinase inhibitor, in melanoma cell lines
Source: J Transl Med. 2008 Sep 29;6:53. doi: 10.1186/1479-5876-6-53 (PMC2569026; doi:10.1186/1479-5876-6-53)
Supplement: Additional file 2 — Comparison of IC50 concentrations of temozolomide when tested alone and in combination with dasatinib in HT144, Lox-IMVI, Malme-3M and Sk-Mel-28 cells. Standard deviations represent average results of triplicate experiments. IC50 values were compared using the Student's T-test. [file 1479-5876-6-53-S2.doc]

Additional file 2: Comparison of IC_50_ concentrations of temozolomide when tested alone and in combination with dasatinib in HT144, Lox-IMVI, Malme-3M and Sk-Mel-28 cells. Standard deviations represent average results of triplicate experiments. IC_50_ values were compared using the Student’s T-test.

| **Cell Line** | **TMZ alone** | **TMZ with dasatinib** | **p value** |
| --- | --- | --- | --- |
| **HT144** | **359 µM**  ± 53 µM | **227 µM**  ± 53 µM | 0.04 |
| **Lox-IMVI** | **162 µM**  ± 49 µM | **45.5 µM**  ± 13 µM | 0.05 |
| **Malme-3M** | **274 µM**  ± 35 µM | **170 µM**  ± 13 µM | 0.02 |
| **Sk-Mel-28** | **465 µM**  ± 19 µM | **412 µM**  ± 45 µM | 0.17 |
