# Supplementary material for: Preclinical evaluation of dasatinib, a potent Src kinase inhibitor, in melanoma cell lines
Source: J Transl Med. 2008 Sep 29;6:53. doi: 10.1186/1479-5876-6-53 (PMC2569026; doi:10.1186/1479-5876-6-53)
Supplement: Additional file 3 — Combination assays of dasatinib with epirubicin or taxotere in HT144 and Lox-IMVI. [file 1479-5876-6-53-S3.doc]

**a)**

**b)**

Additional file 3: Combination assays of dasatinib (D) with epirubicin (E) or taxotere (T) in a) HT144 and b) Lox-IMVI. Drug concentrations are shown in nM. Error bars represent the standard deviation of triplicate experiments.
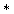
indicates that p ≤ 0.05 for ANOVA comparison of dasatinib alone, chemotherapy drug alone and the combination.
