# Supplementary material for: Preclinical evaluation of dasatinib, a potent Src kinase inhibitor, in melanoma cell lines
Source: J Transl Med. 2008 Sep 29;6:53. doi: 10.1186/1479-5876-6-53 (PMC2569026; doi:10.1186/1479-5876-6-53)
Supplement: Additional file 4 — Effect of dasatinib on cell cycle arrest. Comparing the effect of dasatinib versus untreated cells on the percentage of cells tested in the G1, S and G2/M phases of cell cycle. [file 1479-5876-6-53-S4.doc]

Additional data 4a: Percentage of HT144 cells in the G1, S and G2/M phases of cell cycle. Cells were untreated (control) or treated with variable concentrations of dasatinib. Error bars represent the standard deviation of triplicate assays.

Additional file 4b: Percentage of Lox-IMVI cells in the G1, S and G2/M phases of cell cycle. Cells were untreated (control) or treated with variable concentrations of dasatinib. Error bars represent the standard deviation of triplicate assays.

Additional file 4c: Percentage of Malme-3M cells in the G1, S and G2/M phases of cell cycle. Cells were untreated (control) or treated with variable concentrations of dasatinib. Error bars represent the standard deviation of triplicate assays.

Additional file 4d: Percentage of Sk-Mel-5 cells in the G1, S and G2/M phases of cell cycle. Cells were untreated (control) or treated with variable concentrations of dasatinib. Error bars represent the standard deviation of triplicate assays.
